# Point estimation of certain measures in organizational demography using variable-r methods

**DOI:** 10.4054/demres.2023.49.33

**Published:** 2023-11-16

**Authors:** Michael Lachanski

**Affiliations:** 1 Population Studies Center, University of Pennsylvania, 3718 Locust Walk, Philadelphia, PA, USA.

## Abstract

**BACKGROUND:**

The distribution of job tenure plays an important role in demography, economics, and sociology. Job tenure in a labor market is analogous to age in a population. Demographers have used indirect methods based on variable-r methods to estimate parameters for life table models. The variable-r method can also be employed to estimate the parameters of a job tenure table model that yields the expected length of job tenure and related measures.

**METHODS AND DATA:**

Only two retrospective surveys of current employee tenure lengths and a count of between-survey hires are required to estimate the parameters of a period tenure table using the variable-r method. Tenure-specific sources of decrement allow an analyst to estimate the parameters of multiple-decrement tenure tables and associated single-decrement tenure tables that isolate the proximate contribution of a specific decrement to the job separation process. I illustrate and evaluate the method using publicly available US data.

**RESULTS:**

Variable-r methods generated reasonable parameter estimates: The expected job tenure was 2.48 years at 2002–2004 decrement rates. Multiple-decrement methods can estimate the fraction of employment relationships that end via job displacement. Cause-deleted tenure tables can capture the static effect of eliminating a particular risk to the population of employment relationships.

**CONTRIBUTION:**

Arthur and Vaupel (1984) provide a framework for studying nonstable populations that subsume the variable-r relations that I utilize in this work. Vaupel had an interest in formal demography throughout his life but started his academic career in business statistics. This paper combines those interests.

## 1. Introduction

The length of a new employee’s eventual tenure plays an important role in demography, economics, and sociology (Diebold, Neumark, and Polsky 1997). In labor economics, job search models predict that good matches between employers and employees lead to longer expected employee tenures; economists naturally predict that, at the population level, longer tenures will be associated with workers’ and employers’ satisfaction with current employment relationships (Pries and Rogerson 2022; Molloy, Smith, and Wozniak, forthcoming). Conversely, shorter expected tenures naturally imply higher turnovers and reduced investment in workers by their employers (Molloy, Smith, and Wozniak, forthcoming). For economic sociologists studying the varieties of capitalism, longer expected tenures are utilized as proxy for more coordinated and social democratic systems as in Germany, France, and Belgium. Theory predicts, on the other hand, that market-oriented and liberal systems, such as those in the United States, Canada, and Ireland, will have shorter expected tenures for workers (Hall and Soskice 2001; Witt and Jackson 2016; Roberts and Kwon 2017; St-Denis and Hollister 2023: 21). Finally, family demographers have a long interest in the effect of employment instability on fertility levels that intensified in the wake of the Great Recession (Oppenheimer 1988: 565, 580; Ciganda 2015; Alderotti et al. 2021; Owoo and Lambon-Quayefio 2022).

In these studies, the theoretically interesting estimand is not the average tenure of existing employees or any specific attribute of the tenure distribution at a point in time but the job survival distribution and its evolution over time (Lundberg, Johnson, and Stewart 2021; Baum 2022: 543). However, few government statistical agencies provide tenure-specific job separation data required to directly estimate what organizational demographers refer to as the “mortality rates of jobs” (Stewman 1988: 181). As a result, research on job tenure has tended to rely on snapshot cross-sectional data that can distort the picture by confounding fluctuations in hiring and historical job separation conditions with genuine changes to the job survival distribution over a period (Diebold, Neumark, and Polsky 1997; Heisz 2005: 109–110; Baum 2022: 543). Formal demographers have developed indirect estimation methods that yield period summary measures of mortality conditions when key data are faulty or absent (Preston, Heuveline, and Guillot 2001). This paper adapts one set of indirect estimation methods based on the variable-r equations to the problem of summarizing period job survival. Furthermore, I illustrate how variable-r methods can be used to estimate a number of new measures that isolate the effect of particular decrements on the job separation process.

## 2. Background: Prior research

Job tenure surveys both in the United States and in other developed countries typically measure the length of the current employer–employee relationship, retrospectively (Hyatt and Spletzer 2016; St-Denis and Hollister 2023: 9; Molloy, Smith, and Wozniak, forthcoming). Although in principle an employee can have multiple jobs without changing employers, the literature has often used ‘employee tenure,’ ‘tenure duration,’ ‘employment tenure,’ and ‘job tenure’ interchangeably (Ureta 1992: 324; Diebold, Neumark, and Polsky 1997; Mumford and Smith 2004; Baum 2022; Molloy, Smith, and Wozniak, forthcoming). I will refer to the relationship between an employer–employee involving the regular exchange of wages by an employer for work by the employee as a job or an employment relationship. All jobs created in the same year will be referred to as sharing a job vintage, which plays the same role as the birth year cohort in the formal demography of human populations. I describe the expected length of time that an employee spends with their employer after a hire at current decrement rates as the ‘expected job tenure,’ which I denote by $e_{0}$ (Diebold, Neumark, and Polsky 1997: 218).^2^

### 2.1 Motivation

Recent papers investigating the changing distribution of US tenures as an outcome have tended to regress pooled cross sections of tenure constructed from the Current Population Survey Job Tenure Supplements (CPS JTS hereafter) on discrete time dummies, typically with race or education entering as controls (Farber 2010; Hollister and Smith 2014; Hyatt and Spletzer 2017; Molloy, Smith, and Wozniak, forthcoming). The coefficients generated by the regression approach do not, in general, directly translate into expected job tenures. For instance, a drop in hiring, like that which occurred during the Great Recession, will result in higher mean job tenures at a point in time even if the expected job tenure remains constant (Neumark, Polsky, and Hansen 1999: S33; Heisz 2005: 109–110; Hollister 2011; Copeland 2012; Hyatt and Spletzer 2016, 2017). Similarly, an increase in job separations at high tenures in the distant past would reduce average job tenures today but might not reflect recent tenure-specific job separation rates.^3^ Methods relying on the comparison of snapshot measures of job tenures can both obscure true group differences in employee tenure distributions and generate spurious ones (Heisz 2005: 109–110).

Because changes in the current tenure distribution by itself do not generally reflect expected job tenure, another recent line of research tracks individuals’ job spells in longitudinal panel datasets like the National Longitudinal Survey of Youth (NLSY), the Survey of Income and Program Participation (SIPP), or the Panel Study of Income Dynamics (PSID). In principle, a panel dataset tracking a sufficiently large random sample of individual workers’ hiring and exit dates from employers and the reason for separation would be sufficient to estimate the actual distribution of job tenures over time and all related quantities of interest discussed in this article. In the US context, publicly available panels of employee tenures suffer from shortcomings in either survey design or sample size (Gottschalk and Moffitt 1999: S91–S94; Tamborini and Villarreal 2021; Baum 2022; Molloy, Smith, and Wozniak, forthcoming). The NLSY includes fewer than 25,000 total participants drawn from a dozen birth year cohorts in the 20^th^ century. Each SIPP panel spans only a few years. Gottschalk and Moffitt (1999: S98–S100) note that the PSID’s job tenure question has changed over time, the sample frame for the job tenure question includes only heads of households, and the PSID can identify jobs held about only one year apart.

### 2.2 The demographic approach to estimating employee tenure distributions

In contrast to more recent work relying on the direct inspection of longitudinal panels of workers to estimate cohort expected employee tenures, demographic methods favored by an earlier generation of researchers recovered period conditional expected employee tenures or employee separation rates (Silcock 1954; Lane and Andrews 1955; Hall 1982; Stewman 1988; Ureta 1992; Swinnerton and Wial 1996; Diebold, Neumark, and Polsky 1997; Neumark, Polsky, and Hansen 1999; Heisz 2005; St-Denis and Hollister 2023). The substitutability of detailed longitudinal microdata, as one might obtain from a panel like the SIPP, for a second cross section of job tenures is the major contribution of the formal demographic approach (Preston 1987: 59; Preston, Heuveline, and Guillot 2001; Heisz 2005). Formal demographic methods yield “period measures pertaining to synthetic cohorts” rather than rates pertaining to any one specific cohort (Preston 1987: 41). Synthetic cohort analyses pertain to what would happen if current rates of increment and decrement to the population over a period remained constant (Preston, Heuveline, and Guillot 2001: 38–55).^4^ In exchange for this shift in focus, demographic methods often enable analysts to use large, publicly available cross-sectional datasets to generate synthetic cohort estimates that capture the characteristics of a population (Preston 1987). A comparison of synthetic cohort estimates over time enables an analysis of how the risks a population is subject to have evolved. These synthetic cohort measures are the focus of this article.

Hall (1982: 718) initiates the use of synthetic cohort methods to study US employee tenures using the CPS by estimating the “projected additional time on the job,” which I denote by $e_{x}$, for workers with x years of tenure. Diebold, Neumark, and Polsky (1997) point out that Hall’s method, which relies on comparing ratios of workers with specific employment tenures at specific ages in a single cross section, produces correct tenure distributions only when (1) the rates of labor force exit and entry are constant over time and (2) the tenure distribution is stationary for long periods of time. Subsequent demographic methods for the analysis of labor markets have relaxed both assumptions by using between-survey changes in reported employee tenures to estimate the survival curve prevailing between each survey (Diebold, Neumark, and Polsky 1997; Neumark, Polsky, and Hansen 1999; Heisz 2005; St-Denis and Hollister 2023).

This article contributes a novel demographic approach for estimating the distribution of job survival times in four steps. First, I present a hypothetical tenure table as a model to analyze a population of jobs subject to a decrement process. Second, I introduce the key variable-r relationships and show that they can be used to point estimate the parameters of tenure tables even when longitudinal panel datasets of employment spells and tenure-specific job separations are unavailable. Third, I repeat the first and second steps for the multiple-decrement case. The multiple-decrement analysis introduces new tools to organizational demography in the form of multiple-decrement and cause-deleted tenure tables. These tables yield summary measures that quantify the contribution of distinct decrement types to the period distribution of job survival. I estimate the parameters of a multiple-decrement tenure table and a cause-deleted tenure table. Fourth, I briefly summarize my findings and conclude.

## 3. Job separation as a decrement process: Tenure tables, estimators, and calculations

Jobs – that is, the set of existing employer–employee relationships in a labor market – can be conceived of as a population subject to a decrement process. An employment relationship begins every time a hire occurs. Employee tenure continues (survives) as long as the worker still works for that employer. An employment relationship can end because a worker is fired quits, or dies. The population of jobs sharing a vintage will have its size reduced by these decrement processes until it has vanished. These forces of decrement can be summarized as a single-decrement process that acts to reduce the size of a job vintage over time. Table 1 presents a translation of demographic concepts found in the study of human populations subject to a single-decrement process (mortality) into those found in the study of the population of jobs subject to a decrement process – that is, employer–employee separation. A population’s age structure is analogous to the distribution of job tenure for that population.^5^ Demographic methods applied to the population of jobs yield synthetic vintage summary measures analogous to the synthetic cohort summary measures yielded by demographic methods applied to the population of humans.

### 3.1 Using tenure tables to model the single-decrement process for jobs

By conceiving of employment relationships as a population indexed by tenure and vintage in the same way human populations are indexed by age and birth cohort, researchers can model the survival of a vintage of jobs using a single-decrement tenure table. A single-decrement tenure table is a nonparametric model of the job separation process that describes the size and distribution of survival times of a (potentially hypothetical) member of a vintage. The probability of that member surviving from hiring to x is given by $l_{x}$. The probability of a new employment relationship ending between tenure x and tenure x+n is captured by the dnx parameter. It can be defined as the difference between $l_{x}$ and $l_{x+n}$ as shown in Equation (1):

(1) dnx=lx−lx+n,

where n may be different for different values of x. This implies d∞0=l0=1 because all employment relationships end at a finite time in the tenure table model. Let the tenure-x-specific probability of a job surviving over length n from x to x+n be given by pnx. In the tenure table model, the variable pnx can be computed from the $l_{x}$ column:

(2) lx+nlx=pnxand1−pnx=qnx,

where qnx is the probability of a job ending in an interval from x to x+n. Let Lnx be the expected number of job-years that a new hire will spend in the tenure interval from x to x+n. Then, Lnx can be decomposed as in Equation (3):

(3) Lnx=n⋅lx+n+dnx⋅anx,

where anx captures the expected number of years in the tenure interval from x to x+n for an employment relationship ending in that interval. The expected number of additional years on the job for a worker with tenure x is given by

(4) ex=∑a=x∞Lnalx⇒e0=∑a=0∞Lnal0.

Equations (1) through (4) are the minimal formulae required to define the tenure table model. The variable-r method’s target estimand is Lnx. With limited additional assumptions, the $l_{x}$ curve can be computed using the estimated Lnx parameters and the radix $l_{0}$. All remaining variables in the model are then computed using (1) through (4).^6^ Demographic methods used to obtain life table parameters do not usually estimate Lnx first, but no special difficulties arise from starting with Lnx and $l_{0}$ and computing the remaining variables.

In the study of human populations, analysts sometimes conceptualize $l_{x}$ as the number of survivors reaching exact age x from a hypothetical population of size $l_{0}$ at birth.^7^ A common value for the radix is 100,000. Note that when conceptualizing $l_{x}$ this way, the rest of the human life table columns also change in their interpretation. For instance, Lnx is the expected number of person-years lived in the interval from x to x+n for one newborn person when the radix is 1. When the radix is 100,000, Lnx is the number of person-years lived in the interval x to x+n for a hypothetical birth cohort of 100,000. The vintage tenure table captures the survival experience of a single member of a particular vintage. The population of jobs in a country consists of the sum of jobs across all vintages. A period tenure table summarizes the survival conditions that apply to a member of the population over a time interval from $t_{1}$ to $t_{2}$. A period life table projects the survival experience of a synthetic cohort of people when the population is homogeneous and sufficiently large. A period tenure table projects the survival experience of a synthetic vintage of jobs under the same conditions. If period rates persist indefinitely, then the tenure table will produce an accurate forecast of the distribution of eventual tenure for an arbitrary (and sufficiently large) vintage of jobs. I conceptualize $l_{x}$ as the survival curve for an individual employment relationship for the remainder of this article.

In Table 2, I present a period tenure table model with $l_{x}$ tracking the probability of an employment relationship surviving to x. I omit the left subscript n when all intervals are equal to one year. The columns of Table 2 labeled ‘Selected single-decrement columns’ present the variables $L_{x},\;l_{x},\;d_{x},\;p_{x}$, and $e_{x}$ of a hypothetical period single-year tenure table for the US labor market. From the single-decrement columns of Table 2, one can make numerous statements about the survival time of a hypothetical job if job separation conditions persist. For instance, the mean length of a job for a newly hired worker is 1.5 years $\left(=e_{0}\right)$. That worker’s job has a 68% $\left(=1-\frac{l_{1}}{l_{0}}\right)$ chance of ending before the first year. Conditional on making it to 1 year of tenure, the worker has a 56% $\left(p_{1}\right)$ chance of making it 2 or more years of tenure.

### 3.2 Introducing nonstable population relations

Let N(x,t) be the number of jobs reaching exact tenure x at time t. Each job cohort reaching tenure x at time t is subject to a tenure-specific job separation hazard function μ(x,t), sometimes called a force of decrement, that can change both over the tenure of a job, x, and over time t. Bennett and Horiuchi (1981: 220) show that at any given point in time t, the sizes of a closed population at pairs of tenures x and y are related via the fundamental nonstable population equation

(5) $$N\left(x,t\right)=N\left(y,t\right)\;\text{exp}\;\left\{-\int_{y}^{x}\left[r\left(a,t\right)+\mu \left(a,t\right)\right]\text{da}\right\},$$

with r(a,t) as the growth rate of the number of jobs in the tenure interval from a to a+da in the period ranging from t to t+dt.^8^ Equation (5) has come to be called a ‘variable-r’ relation because it allows for the growth rates at time t,r(a,t), to vary with tenure a.^9^ Tenure-specific growth rates can vary in a population of jobs because of changes in hiring rates or tenure-specific job separation rates that affect particular vintages. As long as the integral on the right-hand side exists, Equation (5) pertains to any closed population of jobs at a moment in time rather than a single vintage. The decrement in this case includes r(a,t), the contemporaneous tenure-a-specific growth rate. The equality in (5) says that the tenure-specific growth rate affects the counts reaching a specific tenure in the same way that a hazard does (Preston 1987: 43). The relationship in (5) can be rewritten to obtain the probability of a job’s survival from y to x in the population at time t, denoted by px−yy(t), as follows:

(6) N(x,t)=N(y,t)exp−∫yxr(a,t)daexp−∫yxμ(a,t)da=N(y,t)exp−∫yxr(a,t)dapx−yy(t).

Setting y=0, note that N(0,t)=B(t) is the number of births at time t. One can rewrite the equality in (6) as

(7) $$N(x,t)=B(t)\;\text{exp}\;\left\{-\int_{0}^{x}r(a,t)\text{da}\right\}p(x,t),$$

where p(x,t) is the probability of a job surviving from hiring to tenure x at the decrement rates of time t. This equation shows that the number of jobs at a specific tenure at time t can be expressed in terms of the number of hires in that period, the job survival function prevailing in that period, and the set of tenure-specific growth rates in that period (Preston, Heuveline, and Guillot 2001: 173).

Using (7), isolate p(x,t) to obtain

(8) $$p(x,t)=\frac{N(x,t)}{B(t)}\;\text{exp}\;\left\{\int_{0}^{x}r(a,t)\text{da}\right\},$$

with the left-hand side term of Equation (8) being exactly the job survival curve from tenure 0 to x at time t. The key idea in (8) is simple: Current tenure-specific employment growth reflecting past shocks to hires and job separations distorts the current tenure structure relative to what it would be if current decrement and hiring rates persisted indefinitely. In particular, the employment tenure of a population of jobs would be exactly that of a stationary population produced by the current decrement function if the r(a,t) terms were zero at all tenures a.

#### 3.2.1 Point estimating a single-decrement tenure table with variable-r methods

Equation (8)’s right-hand side includes hires at time t, an integral of the tenure-specific growth rate up to duration x, and a count of the workers reaching exact tenure x at time t. These quantities have (in principle) observable equivalents in discrete time and discrete tenure data. These facts imply that an analyst can use a discretized form of Equation (8) to estimate life table parameters when the underlying population’s growth rate has varied over time (Arthur and Vaupel 1984: 220, 225; Preston 1987; Preston, Heuveline, and Guillot 2001).^10^ By analogy, variable-r methods enable point estimation of the parameters of a tenure table from job tenure and hiring data.

Let a measurement of a closed population of jobs indexed by tenure be taken at time $t_{1}$ and a second measurement of the same population be taken at $t_{2}$ with $t_{2}>t_{1}$.^11^ I use the method proposed by Preston, Heuveline, and Guillot (2001: 184–187) that takes in cross-sectional counts of the population by duration at time $t_{1}$ and $t_{2}$ to construct a type of midpoint population for which a discretized variation of Equation (8) holds. I use asterisk superscripts to refer to the variables characterizing this midpoint population. The survival conditions that apply to the midpoint population are used to estimate those that prevail in the interval between $t_{1}$ and $t_{2}$. The estimator L^nx of the period tenure table’s Lnx parameter will be the Lnx* that is estimated for a member of the midpoint population using Equation (8).

The midpoint population’s hiring, $B^{*}$, can be estimated by summing hires over the period from $t_{1}$ to $t_{2}$ divided by the length of the interval separating the two cross sections $t_{2}-t_{1}$. Let Nnx(t) be the size of the population with tenure from x to x+n at time t. Then, the midpoint population’s Nnx* is estimated by the geometric mean of the population in the tenure range from x to x+n between $t_{1}$ and $t_{2}$:

(9) Nnx*=Nnxt2⋅Nnxt1.

The discretized rnx* is the growth rate employment of workers with tenure x to tenure x+n for the midpoint population. If the tenure-specific growth rate changes linearly over the interval from $t_{1}$ to $t_{2}$, then the exact tenure-x-specific growth rate for the midpoint population is given by

(10) rnx*=lnNnxt2Nnxt1t2−t1,

which is the mean growth rate over the entire interval. Suppose that one is working with n year tenure groups. Then, our discretized analogue to the integral in (8) for the midpoint population is given by

(11) Sx=n2rnx*+1[x≥n]⋅n⋅∑a=0x−nrnx*.

The general idea of the $S_{x}$ function, which can be adapted to intervals of arbitrary size, is that $S_{x}$ sums each rnx* multiplied by the length of the interval from x to x+n up from 0 to the midpoint of the tenure interval under consideration. The outputs of (9) and (11) are input into the following formula, which yields the expected number of years that a member of the midpoint population would live from x to x+n,Lnx*:

(12) Lnx*=Nnx*B*expSx=Lnx^,

where the quantities in the middle of the two equalities in (12) are observable (in principle). The right-hand side variable Lnx^ indicates that Equation (12) estimates Lnx for the period tenure table prevailing from $t_{1}$ to $t_{2}$.

The inputs required for estimating the tenure table are then simply counts of employees grouped into appropriate x to x+n tenure intervals at $t_{1}$ and t2,Nnxt1 and Nnxt2, and mean annual between-survey hiring $B^{*}$. From counts of employees grouped by job tenure, one computes the midpoint population quantity Nnx* using Equation (9) and the mean tenure-specific growth rates using Equation (10). Equation (12) yields Lnx* for each interval x to x+n in the between-survey period. The midpoint population’s Lnx* is then the estimator L^nx for an Lnx parameter from a tenure table that prevails in the between-survey period.

Researchers have proposed a number of domain-specific rules to estimate $l_{x}$ from Lnx (Preston, Elo, and Stewart 1999: 172; Li and Gerland 2013: 3). I use the estimator

(13) lx=n2n1+n2Lx−n1^n1n1+n1n1+n2Lx−n2^n2n2,

which is exact when (i) $l_{x}$ is linear in the interval $\left(x-n_{1},x+n_{2}\right)$, and (ii) there is no net migration at a given x. I provide a derivation of Equation (13) in Appendix A. All other tenure table variables can be computed from the Lnx and $l_{x}$ columns.

#### 3.2.2 Digit preference and heaping in employee tenure data

The first advantage of variable-r methods is they do not require an analyst to have data grouped into tenure intervals that are a multiple of the time interval between surveys (Preston and Coale 1986: 463–464; Preston, Heuveline, and Guillot 2001: 186). The ability to estimate tenure table parameters even with tenure intervals that are not a multiple of the time interval between surveys turns out to be valuable because it enables an analyst to flexibly abridge the tenure data. Abridged data consists of data grouped into intervals greater than 1 – for example, into the intervals [0,1), [1,5), and all multiples of 5 thereafter. One might wish to abridge tenure intervals because US job tenure survey respondents tend to overreport report tenures ending in 5 or 10 (Ureta 1992; Swinnerton and Wial 1995; Diebold, Neumark, and Polsky 1997: 208, 212–213; Neumark, Polsky, and Hansen 1999). The visual signature of this digit preference bias is ‘heaping,’ which refers to the tendency for a histogram of durations to spike at particular intervals (e.g., 5, 10, 15, and so on). Heaping in human population data can yield incoherent life table parameter estimates that imply an upward sloping survival curve (Preston and Bennett 1983: 103–104; Coale, John, and Richards 1985: 622; Stupp 1986: 53–54; Preston 1987: 45). Similarly, an analysis of US employment tenure data that does not account for heaping can also result in an upward sloping estimated survival curve (Ureta 1992; Swinnerton and Wial 1995; Diebold, Neumark, and Polsky 1997: 217).^12^ By abridging, the analyst can reduce digit-bias-driven heaping by spreading the mass of data near a heap over a longer tenure interval.^13^ For instance, an analyst who believes that respondents have a digit preference for the 5^th^ and 10^th^ years might prefer to work with tenure data binned into five-year groups rather than single-years. I exploit the flexibility of variable-r methods to point estimate the parameters for two single-decrement tenure tables. The first tenure table uses single-year tenure intervals while the second tenure table’s intervals are abridged to [0, 1), [1, 2), [2, 5), [5, 10), [10, 15), [15, 20), [20, 25), [25, 30), [30, 35), [35, 40), and [40, ∞).

#### 3.2.3 Illustrative application of the variable-r estimator to the single-decrement case

In my illustrative application I ask whether the single-decrement tenure tables output by the variable-r method (i) are coherent and (ii) reproduce stylized facts of the US employee tenure distribution reported in Farber (1999). I use two sources of data for calculations in the single-decrement case: the Job Openings and Labor Turnover Survey (JOLTS) and the Integrated Public Use Microdata Series (IPUMS) CPS JTS. From the JOLTS, I use nonfarm, nonseasonally adjusted total hiring counts from January 2002 to December 2003 to estimate $B^{*}$. The biennial IPUMS CPS JTS from 2002 to 2004 is used for obtaining estimates of the size of worker tenure groups Nnx(2002) and Nnx(2004) and of the tenure-specific growth variable rnx*. The self-employed and those younger than 16 are excluded from the CPS JTS.^14^ Further details on the datasets are provided in Appendix B.

Table 3 presents the results of the single-year tenure table for 2002 to 2004 estimated using Equations (9) through (13). Table 4 reports the estimated parameters for the same time period using abridged data.^15^ The radix of both tables is set to 1 so that $l_{x}$ traces a survival probability from hiring to tenure x given the between-survey conditions that prevailed between 2002 and 2004.^16^ For the single-year table, the $l_{x}$ curve is mostly but not always downward sloping. The incoherencies are concentrated near years divisible by 5 and older tenure categories, likely because of digit and recall biases. Observed deviations from a downward sloping survival curve are usually small in absolute terms: No $l_{x}$ was ever more than 2 percentage points away from being downward sloping. The abridged tenure table yields a downward sloping estimated job survival curve. Preston (1987) points out that distortions to the data (likely induced by digit preference) that generate an occasionally upward sloping $l_{x}$ curve often have only small effects on the resulting $e_{x}$ series. Indeed, the single-year and abridged data yield similar expected job tenures of 2.36 years and 2.48 years, respectively. Where comparable, the conditional expected job tenures have a similar shape, although they are of (sometimes substantially) different magnitudes. The $l_{x}$ curve from the single-year table is broadly like that obtained from the abridged table.^17^ Coherence considerations will often favor utilizing abridged data, but whether the abridged or single-year $e_{x}$ series should be preferred will depend on the application.

Can the tenure tables output by the variable-r method reproduce the stylized facts of the US employee tenure distribution reported in Farber (1999)? I briefly review these facts. First, Farber (1999) reports that “most new jobs end early.” Concretely, any credible method for estimating a tenure table should find that more than half of all jobs in the United States will end before the 1-year mark: p(1,t)<0.5. Second, “long-term employment relationships are common.” Ureta (1992: 331) finds that at 1970s and 1980s rates of decrement, between 53% and 57% of jobs at a tenure of between 5 and 10 years could expect an additional 5 years of tenure. Similarly, Ureta (1992) computes that the probability of a job surviving an additional 10 years starting from the tenure of 5 to 10 years is between 30% and 36%. Third, at least initially, the probability of a job separation in an interval declines with tenure: The estimated pnx is greater than the estimated pny when x>y for a large span of the data.

It is easy to use Table 4 to check whether these stylized facts obtain. Indeed, our analysis indicates that at the rates pervading from 2002 to 2004, around 68% of all employment separations end before the first year. The first fact holds. Results comparable to those obtained by Ureta (1992) can be obtained via compact expressions in the abridged tenure table. Using estimated $l_{x}$ column in the abridged tenure table, I obtain $\frac{l_{10}}{l_{5}}=0.57$ and $\frac{l_{15}}{l_{10}}=0.64$ and $\frac{l_{15}}{l_{5}}=0.36$. Similar quantities are obtained using the single-year tenure table. These quantities are reasonably close to those obtained by Ureta (1992) using cross-sectional tenure data from 1978 to 1983. The second fact also holds. Finally, I investigate job separation probabilities. It is straightforward to verify in the abridged case that the pnx series rises from x=1 until it peaks between tenures 15 and 20 and then falls.^18^ Overall, the variable-r method, combined with abridging, enables the estimate of coherent tenure table parameters. The synthetic vintage summary measures in Table 4 reproduce key features of the US employment tenure distribution.

### 3.3 Using tenure tables to model the multiple-decrement process for jobs

Up until this point, I have treated job separation as a single-decrement process. Single-decrement processes can often be conceptualized as multiple-decrement processes. For instance, the mortality process for humans can sometimes be decomposed into specific causes of death. For the set of employment relationships in a labor market, let all K exhaustive and mutually exclusive decrements together constitute an additive process so that the total force of decrement from causes i=1,…,K can be written as

(14) $$\mu (x)=\mu^{1}(x)+\mu^{2}(x)+\cdots +\mu^{K}(x),$$

which captures the fact that at time x, job separations can occur for several reasons and that the relative contribution of each decrement to job separation can vary over the tenure of a job. The standard derivation of (14) assumes that forces of decrement are independent in the sense that the elimination of $\mu^{i}(x)$ has no effect on the magnitude of $\mu^{j}(x)$ for all j≠i (Keyfitz and Caswell 2005: 42–43). I assume that forces of decrement are independent in this sense for the remainder of the paper.

A given force of decrement can typically be broken down further. Consider the case of job displacements. Brand (2015: 360) defines job displacement as “A specific form of involuntary job loss that does not include workers being fired or termination for health reasons; it is reserved for involuntary job separation that is the result of economic and business conditions that are largely beyond the control of the individual and thus presumably less governed by worker performance.”

The CPS lists six possible causes of job displacement: (1) an employee’s plant or company can close, (2) there might be insufficient work at a still open plant or company, (3) a position or shift might have been abolished at a still open plant or company, (4) a seasonal job might have been completed at a still open plant or company, (5) a self-operated business might have failed, or (6) something else might have led to job displacement. If i consisted of the force of job displacement and −i consisted of all other employee separations, then Equation (14) could be rewritten as

(15) $$\mu (x)=\mu^{i}(x)+\mu^{-i}(x)=\mu^{i_{1}}(x)+\mu^{i_{2}}(x)+\cdots +\mu^{i_{6}}(x)+\mu^{-i}(x),$$

where the leftmost equality in Equation (15) captures the simplest possible multiple-decrement process. Using the six reasons for job displacement in the CPS, I rewrite the force of decrement from job displacement $\mu^{i}(x)$ in the rightmost equality of (15) as the sum of six independent forces of decrement: $\mu^{i_{1}}(x)+\mu^{i_{2}}(x)+\cdots +\mu^{i_{6}}(x)$. This latter expression captures the contribution of each potential cause of job displacement in a more complex multiple-decrement framework.

The single-decrement tenure table model can be extended to incorporate the multiple-decrement case described in (14) and (15) in two different, complementary ways: (i) a multiple-decrement tenure table and (ii) a cause-deleted tenure table. For human populations, a multiple-decrement life table models the probability of a human dying from a particular cause of death from age x to x+n at period rates. This is the first way of modeling a multiple-decrement process. The second way of modeling a multiple-decrement process is to focus on the arithmetic effect of eliminating a specific source of decrement to the population. For human populations, a cause-deleted life table models what the single-decrement life table would be if a particular cause of death was eliminated. Analogously, a multiple-decrement tenure table models the probability of a new job ending via a particular cause of separation from tenure x to x+n at current decrement rates. A cause-deleted tenure table models what the single-decrement tenure table would be if a particular cause of employer–employee separation was eliminated. The multiple-decrement and cause-deleted tenure table models provide different ways of isolating the quantitative effect of a particular proximate cause of job separation. Variable-r methods suggest new “ways of estimating parameters that were not previously obvious” (Preston 1987: 59) to organizational demographers, including the key parameters for both the multiple-decrement tenure table and the cause-deleted tenure table (Preston, Heuveline, and Guillot 2001).

#### 3.3.1 The multiple-decrement tenure table

In Table 2, I present columns of a multiple-decrement tenure table under the header ‘Multiple-decrement columns.’ The multiple-decrement tenure table model can be constructed from the single-decrement tenure table with a single additional parameter: dnxi. The dnxi parameter captures the probability of a job separation attributable to cause i in the tenure interval x to x+n. The sum of dnxi from x to the highest possible tenure is given by $l_{x}^{i}$. The multiple-decrement tenure table enables the calculation of $l_{x}^{i}$, which is the probability of eventual job separation from cause i for a job with exact tenure x. Therefore, $l_{0}^{i}$ is the probability of a new hire eventually ending from i (e.g., human mortality, quitting, or firing). The parameter dnx−i refers to the sum of all other decrements than i in the interval x to x+n so that dnxi+dnx−i=dnx=lx−lx+n. The hypothetical multiple-decrement tenure table in Table 2 enables an analyst to make numerous statements about the probability that a job that will end via cause i over any tenure interval in the table. Thus, the probability of a new hire ending by cause i in this model is $0.3685\left(=l_{0}^{i}\right)$. The probability that a job with tenure 1 ends by cause i before reaching tenure 2 is $0.1\left(=d_{1}^{i}\right)$.

#### 3.3.2 The variable-r method point estimator for the multiple-decrement tenure table

Variable-r methods supply an estimator for the parameter dnxi and thereby enable calculations using the multiple-decrement tenure table model. First, one can use Equation (7) to estimate the parameters of the multiple-decrement tenure table by multiplying both sides by the force of decrement of interest, $\mu^{i}(x)$, to get

(16) $$N(x)\mu^{i}(x)=B\;\text{exp}\;\left\{-\int_{0}^{x}r(a)\text{da}\right\}\mu^{i}(x)p(x),$$

where I have suppressed the t term in Equation (7) for simplicity. As with Equations (5) through (8), Equations (14) through (16) are general, characterizing labor market dynamics rather than a particular tenure table model.

To connect (16) to data, let the observed decrements at exact duration x be D(x) with decrements attributable to i being $D^{i}(x)$. Notice that $N(x)\mu^{i}(x)=D^{i}(x)$. If a tenure table held at tenure x, then the number of decrements from cause i, $d^{i}(x)$, at tenure x would be $p(x)\mu^{i}(x)$. Plugging $p(x)\mu^{i}(x)=d^{i}(x)$ into Equation (16) yields the decrements from displacements in the multiple-decrement life table in Equation (17) below:

(17) $$d^{i}(x)=\frac{D^{i}(x)}{B}\;\text{exp}\;\left\{\int_{0}^{x}r(a)\text{da}\right\},$$

where the key intuition of (17) is the same as for Equation (8). An analyst must inflate (or deflate) the number of exits from a particular source of decrement i to adjust for tenure-specific employment growth between surveys. In the continuous case, the probability that a new job will eventually be destroyed via displacement can be found by setting x in the integral in (17) to infinity.

If (17) is discretized, one obtains

(18) dnxl^=Dnix*B*expSx,

where $S_{x}$ and $B^{*}$ take on the same meanings in Equation (12) and Dnix* is a count of the number of employment relationships lost from cause i from tenure x to x+n that occur for the midpoint population. The only additional input needed to go from the single-decrement tenure table to the multiple-decrement tenure table is Dnix*. It is typically difficult to get a plausible Dnix* estimate because appropriate tenure-specific decrement data are not usually available at the midpoint. An alternative is to estimate Dnix*B* as the count of employment relationships lost from cause i from tenure x to x+n that occur over the entire between-survey period divided by the total number of entries into the population over the between-survey period. When this substitution is made, the right-hand side of (17) is comprised only of (in principle) observable quantities. Since i was chosen arbitrarily, this procedure can be repeated for all decrements in Equation (14) for which an analyst has tenure-specific separation counts to generate estimates dnxl^ of the multiple-decrement tenure table. No explicit data on other sources of decrement is needed. Instead, the contribution of other sources of decrement – that is, the effect of the $\sum_{j}\mu^{j\neq i}(x)$ terms in (14) – is inferred from the inability of cause i combined with tenure-specific employment growth to completely account for the employer–employee separations occurring between surveys.

#### 3.3.3 Illustration of the variable-r method in the multiple-decrement tenure table case

Point estimating the key parameter of the multiple-decrement tenure table requires data containing tenure-specific decrements from the between-survey period. Job displacements are the only tenure-specific source of decrement consistently reported in the IPUMS CPS (Flood et al. 2022). Specifically, the 2004 IPUMS CPS Displaced Worker Supplements (DWS) contains counts of workers who had been displaced from their jobs in the last three years and the number of years of tenure accumulated by the worker at the job they were displaced from (Flood et al. 2022). I include all workers indicating that they had been displaced in the last two years, which corresponds to those workers displaced in the 2002 and 2003 period (the same period for which the single-decrement tenure table was estimated). I exclude self-employed workers, and workers under age 20 are not included in the 2004 CPS DWS. Further details on the DWS are available in Appendix B. I conceptualize the job separation process as arising from a total force of decrement μ(x) that can be divided into the force of decrement at time x due to job displacements, $\mu^{i}(x)$, and all other sources of decrements, $\mu^{-i}(x)$, as in Equation (15). Then, in Table 5, I use Equation (18) and actual tenure-specific job displacements from the 2004 CPS DWS to estimate dnxl^ and construct the multiple-decrement table for job displacements.^19^ Each interval in Table 5 tracks the probability of an employment relationship ending by displacement in that interval. At 2002–2004 rates, a new employment relationship has an 8% chance of ending in a job displacement.^20^

#### 3.3.4 The cause-deleted tenure table

What would happen if $\mu^{i}(x)$ in Equation (15) was set to 0? A tenure table estimating the counterfactual world in which only one cause of decrement is operating is called an associated single-decrement tenure table (ASDTT hereafter). The ASDTT isolates the effect of a particular force of decrement, in this case, $\mu^{-i}(x)$. Because the ASDTT for cause −i was constructed from deleting a cause (i), it will be called a cause-deleted tenure table.

Table 2 presents an example of the type of columns that could appear in the cause-deleted tenure table under the header ‘Cause-deleted columns.’ The quantities with a superscripted * in front come from an ASDTT. In Table 2, cause i was deleted. In the column pn*x−i, the probability of a job surviving from exact tenure x to exact tenure x+n after eliminating the cause of decrement i is computed. The e*x−i column computes the expected job tenure conditional on reaching x years of tenure after eliminating the cause of decrement i. As with the multiple-decrement tenure table, this model enables an analyst to make numerous interesting statements. For instance, eliminating the cause of decrement i would increase the probability of a new hire making it to 1 year on the job by 40 percentage points. In a world in which i was eliminated as a cause of decrement, an employee could expect to work for 3.15 years after one year on the job. The cause-deleted tenure table requires the computation of both pn*x−i and a measure of the mean number of years spent in the interval x to x+n by a job that reaches exact tenure x but ends before tenure x+n.

When cause i is eliminated, the cause-deleted tenure table parameter for the number of years spent in the tenure interval from x to x+n for an employment relationship that ends in that interval is denoted by an*x−i.^21^

#### 3.3.5 The variable-r method point estimator for the cause-deleted tenure table

A number of methods exist for constructing cause-deleted life tables. For the cause-deleted tenure table, I use the solution proposed by Chiang (1968), which is that the force of decrement function from cause i is proportional to the force of decrement function from all causes combined in the tenure interval from x to x+n. Suppose a third party intervenes to delete cause i as a force of decrement from the process summarized in Equation (15). Then, the only remaining force of decrement from Equation (15) is $\mu^{-i}(a)$:

(19) μ−i(a)=R−iμaforx≤a≤x+n⇒pn*x−i=pnxR−i,

where R−i=Dnx−iDnxi+Dnx−i. But R−i=Dnx−iDnxi+Dnx−i=dnx−idnxi+dnx−i. Thus, our previous calculations in Equation (18) yield a straightforward plug-in estimator for $R^{-i}$:

(20) dnx−i^dnxl^+dnx−l^=R−l^⇒pn*x−i=pnxR−l^,

where all that is required to estimate (20) is the single-decrement tenure table (to get pnx) and a tenure-specific source of decrement in the between-survey period (to get $\hat{R^{-l}}$). Years spent with an employer in an interval for those employee–employer relationships that ended in that interval from cause −i before the interval’s end can be filled in using the following estimator (Preston, Heuveline, and Guillot 2001: 84):

(21) an*x−l^=n+R−l^qnxqn*x−ianx−n,

where $\hat{R^{-ı}}$ and qn*x−i=1−pn*x−i are obtained in Equation (20), and qnx is obtained using Equation (2). Values for the anx variable were discussed in connection with the single-decrement table. Together, Equations (18) through (21) are sufficient to obtain the cause-deleted ASDTT when a particular cause of decrement has been eliminated (Preston, Heuveline, and Guillot 2001: 82–84). The cause-deleted ASDTT requires the exact same data inputs as the multiple-decrement tenure table: a source of tenure-specific decrements over the between-survey period and a single-decrement tenure table.

#### 3.3.6 Illustrative application of the variable-r method to estimating ASDTT parameters

In Table 6, I use Equations (18) through (21) to obtain a tenure table in which job displacements are eliminated as a cause of job separation.^22^ The cause-deleted tenure table indicates that the effect of job displacements on expected employee tenure and conditional expected employee tenure is substantial. At the rates prevailing in 2002–2004, eliminating displacements would increase an employee’s expected tenure by 35% (from 2.48 years to 3.34 years) with even greater increases at higher levels of tenure.

How can eliminating displacements have outsize effects on expected job tenure when any given employment relationship has only a modest chance of ending via displacement? There are two contributors to this. First, most employment relationships end via non-displacement sources of decrement in the first year. There is simply not much opportunity for displacements to have a sizable influence on how an employee exits via their role at higher tenures of the tenure table because little of the probability mass of the job survival distribution function lies at higher tenures. If all employment relationships after the first year of tenure ended via job displacement, displacements would still end only about 34% (= 32% + 2%) of all jobs created at the rates prevailing between 2002 and 2004. On the other hand, job displacements constitute between 20% and 30% of all separations in the intervals ranging from 2 to 35 years of tenure. Eliminating these displacement-driven decrements generates a sizable increase in conditional expected job duration at these higher tenures that feeds through the tenure table to lower tenures. Second, in contrast with the human life course, the hazard faced by a job is approximately constant or even decreasing over long spans. Thus, the elimination of a decrement generates a larger population that would survive to tenures at which job separation is unlikely. For humans, the gains in life expectancy arising from the elimination of a particular cause of death after infancy are reduced from the fact that the larger ensuing population would face increased hazards at every interval as they aged.

The tension between the summary measure produced by the multiple-decrement tenure table (that a given job has only a small probability of ending via job displacement) and the summary measure produced in the cause-deleted tenure table (that eliminating job displacements would lead to large gains in expected job tenure at hiring) highlights how the multiple-decrement tenure table model and cause-deleted ASDTT model supply two complementary measures for quantifying the proximate contribution of a source of decrement to the job separation process.

## 4. Discussion

This paper extends the classic single-decrement tenure table model to the multiple-decrement case via the multiple-decrement tenure table model and ASDTT model (Silcock 1954; Lane and Andrews 1955; Stewman 1988). These new models supply summary measures of the job separation process that can quantify the proportion of employment relationships that will end via a particular decrement and the contribution of that decrement to maintaining the current level of expected employee tenure. The ‘new’ but in fact quite old formal demographic models presented here open fresh questions for labor market analysts, such as, How does the effect of a given decrement vary over time and by group? and What are the proximate causes of that variation? Future work might profitably explore these questions by pooling populations and sources of decrement from multiple administrative datasets and surveys.

The conception of the set of employment relationships as a nonstable population subject to decrement enables this paper to make two additional contributions. First, this paper demonstrates how variable-r relations can be used to estimate the parameters of single-decrement tenure tables, multiple-decrement tenure tables, and ASDTTs. Second, this paper applies the proposed estimators to cross-sectional US data from 2002 to 2004. Key parameters were estimated for single-decrement (single-year and abridged) tenure tables, a multiple-decrement tenure table yielding the probability of a job ending in displacement at period periods, and a cause-deleted tenure table quantifying how job displacements sustain the current conditional expected duration of a job. The parameters of the abridged national tenure table were both coherent and consistent with stylized facts of US labor markets documented in previous research (Hall 1982; Swinnerton and Wial 1995, 1996; Farber 2010; Hollister 2011; Hollister and Smith 2014).

This article has focused exclusively on point estimation of tenure table parameters using publicly available datasets. Future work should consider the application of bootstrapping or Bayesian statistical inference to the parameters of tenure table models proposed here. Confidence intervals for the expected duration of a job or the posterior probability distribution of a job ending via displacement would facilitate inference on both group differences and population trends in these summary measures.^23^

## 5. Conclusion

Formal demographers often suggest the application of demographic methods to study the labor force (Keyfitz 1985: 53, 162; Preston, Heuveline, and Guillot 2001: 68, 208; Keyfitz and Caswell 2005: 47, 216, 247–249, 506). This article has taken up some of these suggestions. Proceeding along lines similar to Preston (1987), who applied nonstable population relations to family demography, I demonstrated that variable-r methods can be used for estimating the “mortality rates of jobs” and new summary measures in organizational demography (Stewman 1988: 181). Social scientists can use these new summary measures to study group differences in job market experiences and the evolution of the job market over time. Prior to the early 2000s, however, the combination of high quality, regularly reported employee tenure data and a dataset of regularly reported hiring counts required for estimating the parameters of single- and multiple-decrement tenure tables using variable-r methods did not exist for the United States. In general, formal demographic methods have receded from empirical social science research as longitudinal microdata has become more available (Lee 2001; Schmertmann 2002; Li and Xie 2022). This article presents an example in which new data has made formal demographic techniques more valuable rather than less. Demographers might consider looking for cases in which new datasets facilitate the computation of interesting measures from known but previously inestimable formal demographic relations.

## Supplementary Material

DATA ZIP replicability

## Figures and Tables

**Table 1:** Human lives and employment relationships as populations subject to a decrement process

Human population	Jobs
Living human	Job/employment relationship
Mortality	Job separation
Life table	Tenure table
Born	Hired
Age, x	Employment tenure/job tenure/length of service, x
Death	Job separation
Age at death	Completed length of service (Stewman 1988)
Life expectancy, $e_{0}$	Expected job duration/expected job tenure/expected employee tenure, $e_{0}$
Life expectancy at age $x,e_{x}$	Expected job duration at tenure x/ Expected years of remaining service at tenure $x,e_{x}$
x + Life expectancy at age $x;x+e_{x}$	Eventual tenure (Hall 1982); $x+e_{x}$
Birth cohort	Job vintage or hiring year or hiring cohort
Synthetic cohort	Synthetic vintage
Age-specific mortality rate	Tenure-specific separation rate
Survival curve	Job survival curve
Lnx “Person-years lived in an age interval from x to x+n”	Lnx “Job-years worked in a tenure interval from x to x+n “
Causes of death (e.g., tuberculosis, cancer)	Reasons for separation (e.g., job displacement, quitting, mortality)
Multiple-decrement life table	Multiple-decrement tenure table
Associated single-decrement life table	Associated single-decrement tenure table
Cause-deleted life table	Cause-deleted tenure table

*Source*: Author.

**Table 2:** A hypothetical tenure table for single- and multiple-decrement processes

	Job separation as a single-decrement process				Job separation as a multiple-decrement process				
	Selected single-decrement columns				Multiple-decrement columns		Cause-deleted columns		
Tenure x	$L_{x}$	$l_{x}$	$d_{x}$	$p_{x}$	$e_{x}$	$d_{x}^{i}$	$l_{x}^{i}$	p*x−i	e*x−i
0	0.6600	1.0000	0.6800	0.320	1.50	0.2000	0.3685	0.715	3.15
1	0.2500	0.3200	0.1400	0.560	2.61	0.1000	0.1685	0.661	3.15
2	0.1700	0.1800	0.0200	0.880	3.25	0.0150	0.0685	0.909	3.49
3	0.1400	0.1600	0.0400	0.750	2.59	0.0250	0.0535	0.835	2.79
4	0.1050	0.1200	0.0300	0.750	2.29	0.0125	0.0285	0.887	2.23
5	0.0825	0.0900	0.0150	0.833	1.89	0.0100	0.0160	0.887	1.45
6	0.0625	0.0750	0.0250	0.667	1.17	0.0050	0.0060	0.920	0.57
7	0.0250	0.0500	0.0500	0.000	0.50	0.0010	0.0010	0.000	0.00
8	0.0000	0.0000	-	-	-	-	-		
All						0.3685			

*Source*: Author’s calculation using an artificial job market. I omit the $a_{x}$ column for lack of space.

**Table 3:** Single-year tenure table obtained from US data, 2002–2004

Tenure x	$N_{x}$(2002)	$N_{x}$(2004)	2002–2004 Midpoint $N_{x}^{*}$	$r_{x}^{*}$	$\hat{L_{x}}$	$l_{x}$	$p_{x}$	$e_{x}$
0	24,810,008	23,529,820	24,161,437	−0.0265	0.4145	1.0000	0.320	2.36
1	14,218,684	13,065,533	13,629,919	−0.0423	0.2259	0.3202	0.573	6.07
2	8,525,724	8,721,788	8,623,199	0.0114	0.1407	0.1833	0.906	9.36
3	10,893,774	11,920,307	11,395,487	0.0450	0.1913	0.1660	1.028	9.49
4	8,128,907	8,943,589	8,526,523	0.0478	0.1499	0.1706	0.871	8.11
5	7,299,651	8,489,994	7,872,357	0.0755	0.1472	0.1486	0.823	8.31
6	4,472,386	5,203,025	4,823,892	0.0757	0.0973	0.1223	0.754	8.89
7	3,570,413	4,372,746	3,951,267	0.1014	0.0871	0.0922	0.918	10.74
8	3,384,441	3,601,296	3,491,185	0.0311	0.0822	0.0846	0.804	10.67
9	2,118,158	2,303,136	2,208,711	0.0419	0.0539	0.0681	1.247	12.05
10	4,373,394	4,736,348	4,551,254	0.0399	0.1158	0.0849	0.937	9.03
11	1,956,603	1,575,853	1,755,938	−0.1082	0.0432	0.0795	0.599	8.19
12	2,576,288	2,130,144	2,342,619	−0.0951	0.0520	0.0476	0.946	12.77
13	1,731,331	1,817,683	1,773,982	0.0243	0.0380	0.0450	0.835	12.34
14	1,655,914	1,730,475	1,692,784	0.0220	0.0371	0.0376	1.330	13.77
15	2,608,632	2,911,088	2,755,713	0.0549	0.0628	0.0500	0.948	9.61
16	1,405,145	1,351,431	1,378,026	−0.0195	0.0320	0.0474	0.662	8.81
17	1,265,498	1,367,711	1,315,613	0.0388	0.0308	0.0314	1.002	12.28
18	1,225,933	1,385,771	1,303,404	0.0613	0.0321	0.0315	0.800	11.28
19	580,887	770,090	668,831	0.1410	0.0182	0.0252	1.706	12.82
20	2,175,460	2,359,726	2,265,721	0.0407	0.0676	0.0429	1.003	7.09
21	836,313	543,993	674,499	−0.2150	0.0185	0.0430	0.448	5.50
22	943,516	779,628	857,666	−0.0954	0.0201	0.0193	1.017	11.34
23	887,067	845,004	865,780	−0.0243	0.0191	0.0196	0.883	10.12
24	695,319	716,130	705,648	0.0147	0.0155	0.0173	1.312	10.36
25	1,150,009	1,426,718	1,280,913	0.1078	0.0299	0.0227	0.975	7.22
26	570,117	587,884	578,932	0.0153	0.0144	0.0221	0.643	6.05
27	560,993	563,578	562,284	0.0023	0.0141	0.0142	0.931	8.40
28	600,928	463,760	527,907	−0.1296	0.0124	0.0132	0.747	7.96
28	600,928	463,760	527,907	−0.1296	0.0124	0.0132	0.747	7.96
29	315,593	342,269	328,661	0.0406	0.0074	0.0099	1.553	9.40
30	944,621	1,043,759	992,954	0.0499	0.0234	0.0154	1.007	5.57
31	273,621	331,049	300,968	0.0953	0.0076	0.0155	0.532	4.02
32	347,929	329,164	338,416	−0.0277	0.0089	0.0082	0.973	6.64
33	250,519	287,709	268,471	0.0692	0.0072	0.0080	0.739	5.72
34	156,407	173,390	164,679	0.0515	0.0047	0.0059	1.132	6.53
35	295,006	301,491	298,231	0.0109	0.0087	0.0067	0.928	5.07
36	131,894	123,883	127,826	−0.0313	0.0037	0.0062	0.569	4.06
37	100,455	124,997	112,056	0.1093	0.0034	0.0035	0.846	6.09
38	76,266	84,132	80,103	0.0491	0.0026	0.0030	0.812	6.07
39	43,697	77,665	58,256	0.2876	0.0022	0.0024	3.200	6.40
40+	257,597	313,713	284,274	0.0985	0.0133	0.0078	0.000	1.71

*Source*: Author’s calculations using the 2002 and 2004 IPUMS CPS JTS and BLS JOLTS, 2002 to 2003 (Flood et al. 2022). I estimate $B^{*}$ as 57,525,500 and normalize $\hat{L_{x}}$ and $l_{x}$ so that $l_{0}$ is 1. The $p_{x}$ and $e_{x}$ columns are computed from the $\hat{L_{x}}$ and $l_{x}$ columns. I omit the $a_{x}$ and $d_{x}$ columns for lack of space.

**Table 4:** Abridged tenure table obtained from US data, 2002–2004

Tenure x	Nnx(2002)	Nnx(2004)	2002–2004 Midpoint Nnx*	$r_{x}^{*}$	Lnx^	$l_{x}$	pnx	$e_{x}$
0	24,810,008	23,529,820	24,161,437	−0.0265	0.4145	1.0000	0.320	2.48
1	14,218,684	13,065,533	13,629,919	−0.0423	0.2259	0.3202	0.656	6.44
2	27,548,404	29,585,684	28,548,877	0.0357	0.4888	0.2102	0.656	8.74
5	20,845,048	23,970,197	22,353,074	0.0698	0.4808	0.1379	0.567	9.78
10	12,293,530	11,990,502	12,141,071	−0.0125	0.3014	0.0782	0.642	11.10
15	7,086,095	7,786,091	7,427,852	0.0471	0.2011	0.0502	0.705	11.28
20	5,537,676	5,244,481	5,389,085	−0.0272	0.1533	0.0354	0.697	10.32
25	3,197,639	3,384,209	3,289,602	0.0284	0.0939	0.0247	0.667	8.59
30	1,973,097	2,165,071	2,066,856	0.0464	0.0711	0.0165	0.610	7.18
35	647,320	712,169	678,970	0.0477	0.0296	0.0101	0.471	4.71
40+	257,597	313,713	284,274	0.0985	0.0178	0.0047	0.000	3.76

*Source*: Author’s calculations using the 2002 and 2004 IPUMS CPS JTS and BLS JOLTS, 2002 to 2003 (Flood et al. 2022). I estimate $B^{*}$ as 57,525,500. $\hat{L_{x}}$ and $l_{x}$ are normalized so that $l_{0}$ is 1. The pnx and $e_{x}$ columns are computed from the pnx and $l_{x}$ columns. I report the computed anx and dnx columns in Table 6.

**Table 5:** Computation of the probability that an employment relationship will end in a job displacement at various durations, USA, 2002–2004

Tenure X	Estimated number of displacements by duration, January 2002 to December 2004	Growth rate in number of jobs by duration, January 2002 to January 2004	Sum of growth rates from duration 0 to midpoint of interval	Probability that a job will end in displacement at the stated durations at 2002–2004 rates of decrement
0	1,916,839	−0.0265	−0.0132	0.0164
1	1,266,100	−0.0423	−0.0476	0.0105
2	2,446,108	0.0357	−0.0153	0.0209
5	1,228,102	0.0699	0.2129	0.0132
10	471,354	−0.0125	0.3563	0.0059
15	297,174	0.0471	0.4428	0.0040
20	161,431	−0.0272	0.4926	0.0023
25	121,011	0.0284	0.4955	0.0017
30	99,013	0.0464	0.6824	0.0017
35	25,663	0.0627	0.9178	0.0007
40+	25,186	0.0985	1.2835	0.0008
				Sum = 0.0780

*Source*: Author’s calculations using the 2002 and 2004 IPUMS CPS JTS, BLS JOLTS, 2002 to 2003, and 2004 IPUMS CPS DWS (Flood et al. 2022).

**Table 6:** Cause-deleted tenure table (displacements deleted), USA, 2002–2004

Tenure X	dnx	dnxl^	pnx	pn*x−i	anx	an*x−i	e*x−i	$e_{x}^{o}$	e*x−i−exo
0	0.6798	0.0164	0.320	0.329	0.139	0.148	3.34	2.48	0.86
1	0.1100	0.0105	0.656	0.683	0.143	0.159	8.85	6.44	2.41
2	0.0723	0.0209	0.656	0.741	1.039	1.149	11.88	8.74	3.14
5	0.0597	0.0132	0.567	0.643	1.503	1.698	12.63	9.78	2.85
10	0.0280	0.0059	0.642	0.705	1.794	1.929	13.69	11.10	2.59
15	0.0148	0.0040	0.705	0.775	1.612	1.761	13.62	11.28	2.34
20	0.0107	0.0023	0.697	0.753	2.773	2.853	12.06	10.32	1.74
25	0.0082	0.0017	0.667	0.726	1.384	1.526	10.07	8.59	1.48
30	0.0064	0.0017	0.610	0.695	3.230	3.334	8.29	7.18	1.10
35	0.0053	0.0006	0.471	0.510	1.100	1.234	5.45	4.71	0.75
40+	0.0047	0.0008	0.000	0.000	3.763	4.517	4.52	3.76	0.75

*Source*: Author’s calculations using the 2002 and 2004 IPUMS CPS JTS, BLS JOLTS, 2002 to 2003, and 2004 IPUMS CPS DWS (Flood et al. 2022). The anx and dnx columns are taken from Table 4. I normalize $l_{x}$ such that $l_{0}$ is 1. The estimated dnxl^ column is reported on the same scale.

**Figure A-1:**
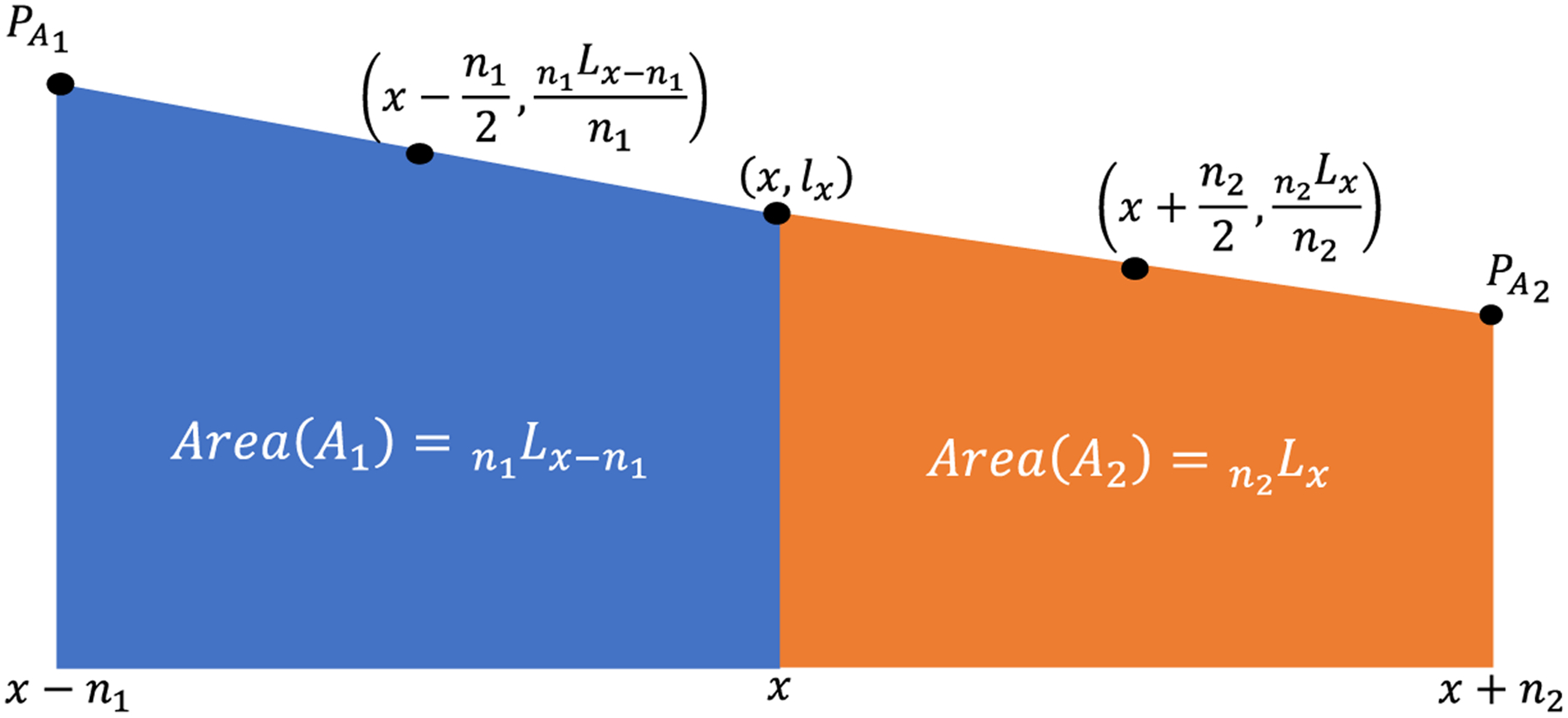
Visualizing the linearization Note: Figure A–1 shows how we derive Equation (13) in the main text from adjacent Ln1x−n1 and Ln2x+n2 terms. If the $l_{x}$ curve is linear over the interval $\left(x-n_{1},x+n_{2}\right)$, then the shape of the area under the $l_{x}$ curve with vertices $\left(x-n_{1},0\right)$, (x,0), $\left(x,l_{x}\right),\;P_{A_{1}}$ will be a right trapezoid, $A_{1}$, with area Ln1x−n1. Similarly, the shape of the area under the $l_{x}$ curve with vertices (x,0), $\left(x+n_{2},0\right),\;P_{A_{2}},\left(x,l_{x}\right)$ will be a right trapezoid, $A_{2}$, adjacent to $A_{1}$, with area Ln2x. The height of each trapezoid at the midpoint is given by x1,y1=x−n12,Ln1x−n1n1 and x2,y2=x+n22,Ln2xn2. The slope m connecting these midpoints is m=Ln1x−n1n1−Ln2xn2n1+n22. Plugging in the points x−n12,Ln1x−n1n1 and $\left(x,l_{x}\right)$ into the point-slope form of a linear equation with slope m yields Equation (13).

## Footnotes

2 Sometimes ‘job tenure’ is used to refer to time spent in a particular job role rather than time spent with a particular employer. Indeed, a literal interpretation of the phrase ‘job tenure’ suggests that it could be less than the time spent with a particular employer. A perhaps more accurate albeit clumsy and rarely used term for concept dealt with in this paper would be ‘expected tenure with a given employer’ or the ‘expected length of the employment relationship.’ The early literature in organizational demography referred to the distribution of eventual tenures as the ‘completed length of service’ and did not make clear distinctions between working for an employer and working in a particular role for an employer (Silcock 1954; Lane and Andrews 1955; Stewman 1988).

3 Only in special (and unrealistic) cases will it be true that the proportion of short-term jobs correctly estimates the fraction of short-term employment arrangements there would be if current conditions persist (Hall 1982; Ureta 1992; Preston, Heuveline, and Guillot 2001: chap. 3; Heisz 2005: 109–110; Lundberg, Johnson, and Stewart 2021). Changes in mean job tenure between two point-in-time estimates do not generally track changes in expected job tenure (Ryder 1975; Heisz 2005: 109–110; Baum 2022).

4 Specific censoring problems present in the analysis of longitudinal panels are not present in the period analysis. For instance, there is no possibility for participants to be lost to follow up since they need to appear in a cross-sectional survey only once.

5 In this framework, particular sectors (e.g., public versus private or various industries) are analogous to human subpopulations that grow at different (potentially negative) rates because they are subject to different processes of decrement and increment. Notice that because the subject in this population is an employer–employee relationship, unemployed workers are not members of the population under investigation.

6 Tenure tables thus provide a unified framework for relating the tenure-x-specific probability of a job surviving from x to x+n and the conditional expected job tenure summary measures utilized in prior research (Hall 1982; Stewman 1988: 181; Diebold, Neumark, and Polsky 1997; Neumark, Polsky, and Hansen 1999: S33; Heisz 2005: 110–111). The tenure table model is nonparametric in the sense that the table makes no strong assumptions about the overall shape of the Lnx and $l_{x}$ functions except that they can be fruitfully studied in tenure-specific intervals.

7 This is sometimes called the “dual interpretation” of $l_{x}$ in that it refers to an individual survival curve and the exact number of people from a given hypothetical birth cohort reaching exact age x (Keyfitz and Caswell 2005).

8 The fundamental nonstable population equation will also hold for a population open to migration if net tenure-specific migration is zero. Nonzero tenure-specific migration can be explicitly incorporated via a more general interpretation of r(a,t), as in Preston (1987).

9 By contrast, for a stable population r(a,t)=r∀(a,t) with r in the set of real numbers. The stable population assumption is unlikely to hold over any substantial length of time for the population of employment relationships because of variation in the hiring and job separation rates.

10 Preston and Coale (1982) are the first to demonstrate how the variable-r equations can be used to approximate the period life table prevailing between population censuses. Arthur and Vaupel (1984: 219, 224) proposed an alternative framework for analyzing nonstable populations, encompassing Preston and Coale’s variable-r equations, and extending them to include various other relationships. Arthur and Vaupel’s (1984: 220) method was intended to be used to compute cohort life tables using historical birth counts. The present work has relied on the variable-r method presented in Preston, Heuveline, and Guillot (2001), which is focused on estimating the parameters of a period life table.

11 A potential weakness of the approach to estimating employment relationship durations utilized in this paper compared to approaches based on longitudinal panels discussed above lies in the handling of immigration. Immigration can generate the appearance of tenure-specific growth (and thus affect estimated job survival probabilities) if immigrants arrive to the United States and retain their employer. This tenure-specific growth, in turn, would bias period job separation probability estimates. I assume that net migration to the United States has no impact on the tenure-specific employment distribution. This can be justified if immigrants arrive to the United States with zero years of tenure and are then hired locally. If new hires were broken out by immigration status, it would be possible to analyze native-born and foreign-born populations separately. Preston (1987) presents an explicit solution to account for this possibility if data on immigration and emigration is available by employee tenure. No US hiring series I am aware of distinguishes hires by nativity. If the native-born and foreign-born are analyzed separately, then it must also be assumed that emigrants do not affect tenure-specific employment growth. This seems reasonable given the discussion in Hall (1982: 719).

12 Heaping can also arise from the fact that some occupations like teachers are hired only at specific time points (Allen, Burgess, and Mayo 2017).

13 An analyst may not wish to alter an incoherent output life table at all if the incoherences are occasional and small, especially if the target measure is an expected duration or conditional expected duration (Preston 1987: 46; Preston, Heuveline, and Guillot 2001: 187). On the other hand, analysts might try to correct for potential biases induced by digit preference either via preprocessing the input data (e.g., via abridging) or postprocessing the life table output of variable-r-based routines (Coale, John, and Richards 1985: 622; Preston 1987; Preston, Heuveline, and Guillot 2001: 168, 194–197; Hobbs 2004).

14 The between-survey period spanning the years 2002 to 2003 (inclusive) is the first period for which all three inputs required for estimating the parameters of the tenure table, two employment tenure distributions and a count of overall hires between them, are available.

15 The fact that the rnx* column varies in sign across tenure intervals in both Tables 3 and 4 motivates the use of variable-r methods because it suggests that tenure-specific employment growth rates are not constant.

16 The mean annual number of hires $B^{*}$ between January 2002 and 2004, the CPS JTS survey date, is required to estimate $e_{0}$. On the other hand, conditional job tenure expectancies starting from $e_{2}$ can be computed directly from the two cross sections without hiring data.

17 Numerous options are available for minimizing divergences in the $l_{x}$ variable between the single-year and abridged tenure tables. Analysts could also estimate $l_{x}$ from the single-year tenure table model and directly input these into the abridged table for computing the rest of the abridged tenure table variables. In this case, (13) reduces to $\frac{\hat{L_{x-1}}+\hat{L_{x}}}{2}$. Another option would have been to estimate the $L_{x}$ column using single-year data and then abridge it when computing the rest of the tenure table parameters.

18 Rising separation probabilities for very high tenures are consistent with Tables 2, 3, and 4 in Diebold, Neumark, and Polsky (1997) and Tables 1, 2, and 3 in Neumark, Polsky, and Hansen (1999). A reduced four-year retention probability necessarily implies a higher hazard rate. Prior work typically studied the population of employee tenures greater than 21 as a single group (Diebold, Neumark, and Polsky 1997: 217; Neumark, Polsky, and Hansen 1999: S59).

19 The linearity of $l_{x}$ and estimates of Lnx are sufficient to identify anx in Table 5‘s multiple-decrement estimate. Another potential tenure table incoherency could arise in the form of a negative anx value, which can occur when data flaws lead to an upward sloping Lnx curve. This incoherency can sometimes be eliminated via abridging to larger intervals.

20 The period probability of job displacement is likely understated because the CPS DWS records up to only one displacement in the past two-year period. If someone has been displaced multiple times in the two-year interval between surveys, only one decrement will be recorded. It may be possible to correct this bias by obtaining between-survey displacement decrements from an alternative source like the SIPP. A full exploration of the size of this bias is outside the scope of this article.

21 Smith (2009: 235–236) argues that cause-deleted life tables are informative about potential outcomes. It is difficult to imagine an intervention that would reduce the hazard of a source of job separation (e.g., job displacements) $\mu^{i}(x)$ to zero without affecting other tenure-specific forces of decrement (e.g., quitting). The cause-deleted tenure table is most defensible as an alternative summary measure of how a particular cause of decrement contributes to the overall job separation process rather than a description of a counterfactual in which the cause was eliminated.

22 For Table 6, I assume an upper bound to job tenures of 45 to ensure consistency with Table 4.

23 To further develop these methods, I have added variable-r life table estimation functions to the demographic code repository DemoTools. I have also implemented a simple variable-r function specifically for estimating tenure table parameters, example data, and a brief tutorial for using the function that can be downloaded as an Rmarkdown file from: https://osf.io/e6ghx?view_only=60583c4730b7495d8240226fa4f3144a.

## Appendix A: Converting Lnx^tolx

### A. 1 Description of the problem

There are few appropriate methods in the actuarial, demographic, and public health literatures enabling the derivation of a plausible $l_{x}$ curve from an Lnx series. For instance, it is conventional to assume that anx=n2 in human populations (Wachter 2014: 59, 75, 154–156). Then, when one has an Lnx series and a radix, one can recursively fill out the rest of the $l_{x}$ series via repeated applications of Equation (3). The assumption that anx=n2 is strongly violated in the tenure table model because it is often the case that, in the first interval, Ln0<n2. For example, in Tables 3 and 4, L10<12 holds, and so a10<12 must also hold. Assuming that a10=12 would lead to an incorrect estimate of $l_{1}$ that propagates through the entire $l_{x}$ series.

With the exception of Preston (1987) and Preston, Heuveline, and Guillot (2001), the published methods on the conversion of Lnx to $l_{x}$ are idiosyncratic and context-specific. For example, Li and Gerland (2013) and Preston, Elo, and Stewart (1999) focus on the Lnx→lx conversion for older populations (although their relations might be useful for younger populations as well). No published methods are appropriate for such a conversion when the length of adjacent intervals is irregular. Nonetheless, Preston (1987) and Preston, Heuveline, and Guillot (2001) describe a conversion formula appropriate for regular intervals that is exact when (i) there is no net migration at a given x, and (ii) $l_{x}$ is linear over the adjacent intervals $\left(x-n_{1},x\right),\left(x,x+n_{2}\right)$ with $n_{1}=n_{2}$ (since the intervals are regular).^24^ I generalize this estimator in Equation (13) to allow for irregular intervals in which $n_{1}\neq n_{2}$. When $n_{1}=n_{2}$, my proposed conversion reduces to that proposed by Preston (1987) and presented in Preston, Heuveline, and Guillot (2001).

### A. 2 Derivation of main text Equation (13)

I recover the slope of a line connecting the midpoints of two adjacent trapezoids with areas Ln1x−n1 and Ln2x, respectively. I visualize this in Figure A–1. This approach turns out to be sufficient to obtain $l_{x}$. Suppose that Ln1x−n1 and Ln2x are the areas of adjacent right-angle trapezoids $A_{1}$ and $A_{2}$, respectively. The height of $A_{1}$ at its midpoint is given by Ln1x−n1n1. The height of $A_{2}$ at its midpoint is given by Ln2xn2. The slope of a line connecting the midpoints of the top legs of $A_{1}$ and $A_{2}$ is m=Ln1x−n1n1−Ln2xn2n1+n22. The base shared by $A_{1}$ and $A_{2}$ has height $l_{x}$. I assume that $l_{x}$ falls on a curve that has slope m from the midpoint of $A_{1}$ at $\frac{n_{1}}{2}$ to $n_{1}$. We can then obtain the height $l_{x}$ by plugging in the ‘data point’ x1,y1=x−n12,Ln1x−n1n1 into the point-slope form of a line equation that $l_{x}$ falls on, yielding the following line equation for $l_{x}$ in y=mx+b form:

lx=y=2n1+n2Ln2xn2−Ln1x−n1n1n1−n12+n1Lx−n1n1,

which reduces to Equation (13) in the main text:

lx=n2n1+n2Ln1x−n1n1+n1n1+n2Ln2xn2.

When $n_{2}=n_{1}=5$, this reduces to

lx=L5x−5+L5x10,

which is the formula given in Preston, Heuveline, and Guillot (2001: 186). If $n_{2}=n_{1}=1$ or $n_{2}=n_{1}=10$, then I obtain the additional formulae presented in Preston (1987: 45). Equation (13) thus pools information from adjacent Lnx terms to generate an $l_{x}$ series. A desirable feature of this approach is that there is no recursion within the computation of the $l_{x}$ series itself. Thus errors in the $l_{x}$ series at low tenures do not necessarily propagate throughout the $l_{x}$ curve.

## Appendix B: Dataset details

### B. 1 Data for single-decrement calculations

The JOLTS hiring series is a monthly set of total nonfarm US hires.^25^ The JOLTS is administered to around 20,000 firms and weighted to be nationally representative. JOLTS excludes agriculture and private household hiring.

As mentioned above, recent research has studied changing employment tenures using longitudinal panel datasets – for example, using the NLSY, PSID, and SIPP (Jaeger and Stevens 1999; Farber 2000; Mouw and Kalleberg 2010; Baum 2022; Molloy, Smith, and Wozniak, forthcoming). Compared to these longitudinal panel data sources, over the time period I am interested in studying, the CPS JTS yields a similar distribution of employee tenures to the PSID while enabling a richer exploration of time, race, sex, public/private status, occupation, and industry with consistently large samples (Gottschalk and Moffit 1999: S93; Wrigley-Field and Seltzer 2020: 13–14; Jaeger and Stevens 1999: S26–S27).^26^ Hyatt and Spletzer (2016) find that the overall tenure distribution reported in the CPS JTS tracked administrative data remarkably well. The CPS JTS question I use to track tenure asks, “How (have/has)(you/name) been working CONTINUOUSLY ((for) fill company name from basic CPS)(as a self-employed person at (his/her/your) present business)(at (your/his/her) main job)(for (your/his/her) present employer?).”^27^

A challenge in these datasets is harmonizing the data across sources for the self-employed. The sampling frame for the JOLTS is primarily drawn from the Quarterly Census of Employment and Wages (QCEW), which excludes all nonincorporated self-employed workers. Incorporated self-employed workers in principle could be included via their participation in unemployment insurance systems at the state level, but not all incorporated entities participate in unemployment insurance systems. I am not aware of research on how well JOLTS covers hiring for self-employed workers in incorporated entities. The CPS JTS distinguishes between self-employed incorporated and self-employed unincorporated workers, allowing these data sources to be harmonized in principle. I drop all self-employed workers in my illustrative analysis for simplicity.^28^

A second potential discrepancy concerns age. The JTS was administered to workers aged 15 and older. The QCEW, from which JOLTS is drawn, includes workers 16 years of age or older. However, because the JOLTS draws on the establishments in the QCEW rather than individual workers, a worker would need to be employed at a firm that employs only workers 15 years of age to have the possibility of being excluded from the JOLTS but included in the CPS JTS. In the single-decrement analysis, I include all JTS workers aged 16 and older, but including workers aged 15 made no difference to the results. Including agricultural workers made minimal difference to the single-decrement or multiple-decrement table results. For simplicity, I included them in the analysis.

### B. 2 Data details for multiple-decrement calculations

For both the JTS and DWS surveys, it is most convenient if the decrements from displacements are measured over the same interval with which the number of job-years worked is computed. The DWS in 2004 contains job displacement decrements from 2003, 2002, and 2001. The between-survey period for the CPS JTS includes 2002 and 2003 but not 2001. Therefore, in the 2004 DWS, I count decrements from displacements occurring only in the previous year (2003) or the year before (2002).

The CPS DWS does not clearly distinguish between displacements from an unincorporated self-employment business relationship and displacements from an incorporated self-employment relationship. Instead, the DWS distinguishes between being displaced from a family-owned self-employed business and a non-family-owned self-employed business. As with the single-decrement estimates, I drop all self-employed workers. While the single-decrement calculations included workers aged 16 and older, the DWS was administered only to workers aged 20 and older. I use the same CPS JTS sample for the multiple-decrement analysis that I use in the single-decrement analysis. The DWS’s exclusion of workers aged 15 to 19 has the effect of reducing the quantitative effects of displacements overall. Distortions in estimates derived from tenure-specific displacement effects are likely limited to jobs and employment relationships that have accumulated less than 5 years of employee tenure.
